# Radiation Recall Reaction: Two Case Studies Illustrating an Uncommon Phenomenon Secondary to Anti-Cancer Agents

**DOI:** 10.7497/j.issn.2095-3941.2012.03.009

**Published:** 2012-09

**Authors:** Su-yu Zhu, Yuan Yuan, Zhen Xi

**Affiliations:** Department of Radiation Oncology, Xiangya Medical College Affiliated Cancer Hospital of Hu’nan Province, Zhongnan University, Changshan 410013, China

**Keywords:** radiation recall, radiotherapy, dermatitis, pharyngitis, docetaxel, case report

## Abstract

Radiation recall phenomenon is a tissue reaction that develops throughout a previously irradiated area, precipitated by the administration of certain drugs. Radiation recall is uncommon and easily neglected by physicians; hence, this phenomenon is underreported in literature. This manuscript reports two cases of radiation recall. First, a 44-year-old man with nasopharyngeal carcinoma was treated with radiotherapy in 2010 and subsequently developed multi-site bone metastases. A few days after the docetaxel-based chemotherapy, erythema and papules manifested dermatitis, as well as swallowing pain due to pharyngeal mucositis, developed on the head and neck that strictly corresponded to the previously irradiated areas. Second, a 19-year-old man with recurrent nasal NK/T cell lymphoma initially underwent radiotherapy followed by chemotherapy after five weeks. Erythema and edema appeared only at the irradiated skin. Both cases were considered chemotherapeutic agents that incurred radiation recall reactions. Clinicians should be knowledgeable of and pay attention to such rare phenomenon.

## Introduction

Radiation recall reaction is an uncommon and unpredictable phenomenon that is characterized by an acute inflammatory reaction confined to previously irradiated areas and triggered by the administration of precipitating systemic agents after the radiation treatment ^[^^1^^-^^4^^]^. Anticancer agents are the most commonly believed causes of radiation recall, but other triggers could include several antibiotics, antituberculosis drugs, and simvastatin ^[^^1^^,^^3^^]^. At the chemotherapeutic departments, most patients are treated by oncology physicians rather than radiation oncologists; thus, radiation recall reactions are easily neglected in clinics, and thereby, underreported in literature. This article reports two cases with photos that depict the corresponding areas of previous irradiation dose distribution and recall reaction.

## Case One

A 44-year-old man was clinically diagnosed with nasopharyngeal carcinoma stage T_2_N_3_M_0_ and IVa as well as pathologically confirmed as having poorly differentiated squamous cell carcinoma. The patient was initially treated with 1 cycle of chemotherapy with paclitaxel 210 mg and cisplatin 120 mg from April 5 to 7, 2010. From April 14 to June 9, 2010, intensity-modulated radiotherapy (IMRT) was delivered with the following treatment plan and target volumes: 70 Gy in 33 fractions (fx) for 57 days for the primary nasopharyngeal lesion; 70 Gy in 33 fx for 57 days for the neck metastatic nodes; and 58 Gy to 61 Gy in 33 fx for 57 days for the other neck areas.

**Figure 1:**
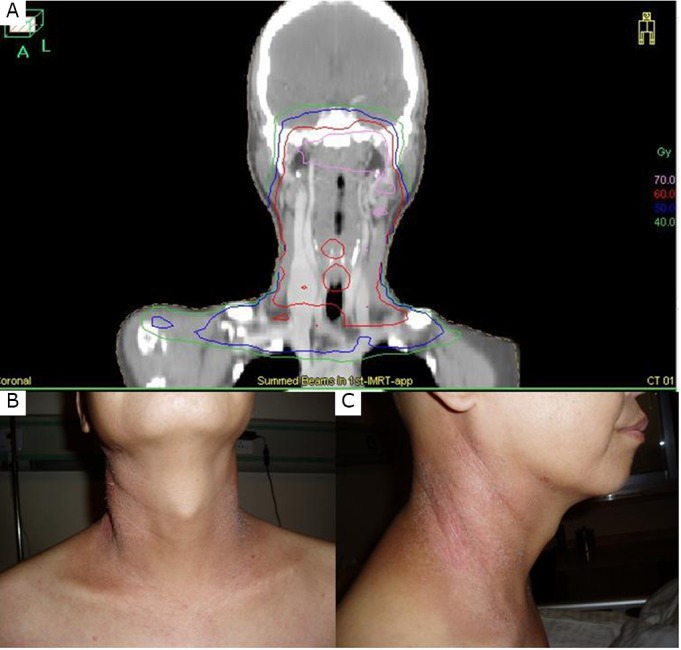
Docetaxel-induced radiation recall dermatitis. A: Coronal display of the dose distribution for case one (nasopharyngeal carcinoma) with IMRT in 2010; B and C: Frontal and lateral views of erythema and desquamation in concordance with areas that received previous radiation dose distribution.

One year after radiotherapy, from June to September 2011, the patient suffered from lower back pain. The entire skeleton bone scintigraphy and lumbar MRI revealed multi-site bone metastases with the most severe bone erosion at lumbar 1. Palliative radiotherapy was delivered for the posterior field ranging from thoracic 12 to lumbar 2 at a dose of 40 Gy in 20 fx for 31 days. Subsequently, chemotherapy with docetaxel 160 mg (95 mg/m^2^) and cisplatin 120 mg (70 mg/m^2^) was administered on October 10, 2011. Five days later, erythema, edema, pururitis, and papules followed by light scaling appeared on both sides of the neck skin. In addition, swallowing pain due to pharyngitis and a symptom of dysphagia manifested during the same period. All of these symptoms occurred in the previously irradiated area (**Figure 1**). Docetaxel-induced radiation-recall dermatitis and pharyngitis were logically inferred. A daily dosage of 10 mg of dexamethasone was intravenously administered. Seven days after chemotherapy, febrile leukopenia with WBC count of 0.4×10^9^ cells/L developed. Cefazolin and granulocyte colony-stimulating factors were prescribed. The WBC normalized 3 days later. The neck skin normalized 8 days after the inception of radiation recall reaction.

## Case Two

In April 2006, a 19-year-old man was initially diagnosed with nasal non-Hodgkin lymphoma and was pathologically confirmed as peripheral T-cell type. Two cycles of CHOP (cyclophosphamide, doxorubicin, vincristine, prednisone) chemotherapy were administered, followed by conventional radiotherapy with the bilateral nasal cavitie tumor dose of 33.4 Gy in 17 fractions. Thereafter, another two cycles of consolidative CHOP chemotherapy were performed.

In November 2011, nasal cavity recurrence was diagnosed and pathologically classified as nasal-type NK/T cell lymphoma. Conventional radiation was delivered, including the areas of nasal and nasopharyngeal cavities, maxillary, and ethmoid sinus, with a dosage of 17.5 Gy in 9 fractions. IMRT with a dosage of 35.2 Gy in 16 fractions was prescribed to the clinical target volume. The total radiation dose calculated for the nasal cavity was 54.8 Gy in 25 fractions. Upon completion of the treatment, CT scan results indicated unconfirmed complete remission. Grade 2 dermatitis, grade 3 nasal mucositis, and grade 2 stomatitis (CTCAE v4.0) were documented during radiotherapy and resolved within one month after the end of radiation.

**Figure 2:**
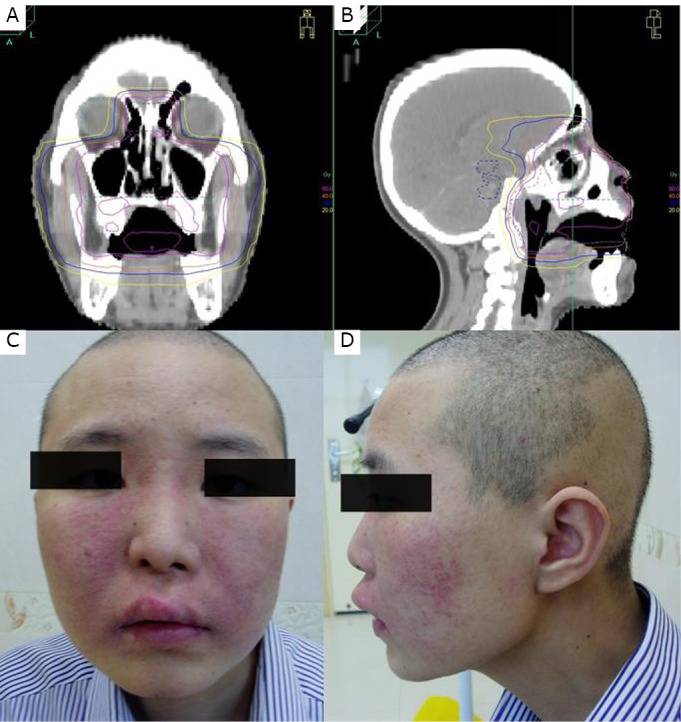
CHOP regimen-induced dermatitis. A and B: Coronal and sagital display of composite isodose distribution of conventional and IMRT for case 2 recurrent nasal NK/T cell lymphoma; C and D: Frontal and lateral photos of erythema and papules demarcated in concordance with areas of previous radiation dose distribution.

After 5 weeks, CHOP chemotherapy was initiated as a consolidative means on March 19, 2012. Three days later, erythema and edema appeared on the skin of the head that previously received the composite isodose distribution of radiation therapy (**Figure 2**). Radiation recall dermatitis was reasonably considered, and the causative link was generally attributed to the agents of CHOP regimen. No special measures were delivered to treat the erythema and edema, which turned into mildly dry desquamation 5 days later and gradually disappeared. Another course of CHOP regimen was administered at the same dosage. Mild erythema and edema reappeared but were similarly resolved without any special treatment.

## Discussion

First described in 1959^[^^5^^]^, radiation recall remains a poorly understood phenomenon. The precise mechanism is unknown, and various hypotheses have been proposed: (1) cytotoxic treatment induces a remembered reaction in the remaining surviving cells; (2) mutation caused by the radiotherapy yields more vulnerable cells that cannot tolerate cytotoxic treatment; and (3) a vascular reaction occurs after radiotherapy^[^^1^^,^^3^^]^.

In literature, most of the reported cases are radiation recall dermatitis. Other reports focused on rare cases, such as inflammatory reactions affecting the lungs, oral mucosa, gastrointestinal system, genitourinary tract, muscle layer, and the central nervous system^[^^6^^]^.

The reported time interval between the end of radiation and the recall reaction ranged from a few days to 15 years^[^^7^^]^. Considering the effects of possible radiosensitization, Camidge and Price ^[^^3^^]^ argued that the interval between radiation therapy and the resultant symptoms should be more than 7 days if radiation recall reactions are to be considered.

The 2 cases reported in this study are both considered radiation recall reactions. In case one, severe swallowing pain and leucopenia prevented endoscopy; hence, no image of mucositis was captured. However, the emerging symptoms of severe swallowing pain and dysphagia as well as the display of high-dose coverage of the mucosa of the retro-pharyngeal wall with previous IMRT could fairly justify the diagnosis of radiation recall mucositis. In case two, the cause of radiation recall dermatitis (RRD) could not be determined, because the agents of cyclophosphamide, doxorubicin, and vincristine have all been reported to cause RRD in literature^[^^8^^-^^10^^]^.

In conclusion, radiation recall reactions are uncommonly detected in clinics and underreported in literature. Clinicians should be vigilant for possible radiation recall reactions upon encountering changes such as the appearance of erythema and edema that are confined to the previously irradiated skin, as well as mucosa during or after chemotherapy.
